# Predictive Value of Preoperative Anatomical and Functional Parameters for Long-Term Visual Outcomes After Full-Thickness Macular Hole Surgery with the Inverted Flap Technique

**DOI:** 10.3390/jcm14248757

**Published:** 2025-12-10

**Authors:** Oskar Lorenc, Krzysztof Safranow, Anna Machalińska

**Affiliations:** 1 First Department of Ophthalmology, Pomeranian Medical University, 70-204 Szczecin, Poland; oskar.lorenc@pum.edu.pl; 2 Department of Biochemistry and Medical Chemistry, Pomeranian Medical University, 70-204 Szczecin, Poland

**Keywords:** full-thickness macular hole, macular hole surgery, inverted ILM flap technique

## Abstract

**Background/Objectives:** The aim of the present study was to identify preoperative functional and anatomical parameters that better predict postoperative best corrected visual acuity (BCVA) after full-thickness macular hole (MH) surgery during long-term follow-up. **Methodology:** Initial visual outcomes, medical history, retinal imaging data, microperimetry and mfERG measurements were collected to characterise functional and morphological macular status. **Results:** Among the study subjects, 22 presented with a BCVA > 0.5, and 20 presented with a BCVA ≤ 0.5 at the final visit. Multivariate regression analysis revealed that a smaller minimum MH diameter (OR = 0.98; 95% CI = 0.87–0.99; *p* = 0.004) and a shorter disease duration (OR =0.11; 95% CI = 0.02–0.53; *p* = 0.005) were predictors of postoperative long-term BCVA > 0.5. Baseline P wave amplitudes in the central ring on mfERG were positively correlated with postoperative BCVA gain (Rs = +0.53, *p* = 0.001). **Conclusions:** Our findings corroborate the significance of hole diameter measurements for postoperative visual outcomes and support the rationale of early intervention.

## 1. Introduction

Full-thickness macular holes (FTMHs) represent a relatively common vitreoretinal pathology, with an estimated incidence of approximately 9 eyes per 100,000 people per year. The condition predominantly affects women, with a female-to-male ratio of approximately 3.3:1 [1]. FTMHs cause visual loss because of full-thickness retinal defects, and the vast majority of cases are idiopathic in origin. Surgical treatment with pars plana vitrectomy (PPV) was first reported in 1991 [2]. Since then, the widespread use of PPV with internal limiting membrane (ILM) peeling and the introduction of the inverted ILM flap technique [3] has resulted in closure rates of approximately 95% [4]. However, despite successful surgical closure and restoration of anatomical shape, postoperative best-corrected visual acuity (BCVA) remains variable, with excellent visual outcomes (BCVA > 0.5) achieved in only approximately 50% of cases [4,5]. In recent years, the focus has shifted from anatomical closure of the hole to achieving excellent visual acuity. Several studies have attempted to identify primary OCT hole morphology and visual acuity status that predict postoperative BCVA [6]. In our study, we evaluated both anatomical and functional preoperative parameters to determine their ability to predict a BCVA > 0.5. While previous studies have investigated structural predictors of surgical success, e.g., integrity of the external limiting membrane, ellipsoid zone or interdigitation zone [7]. To our knowledge, this is the first study to combine detailed preoperative functional assessment with comprehensive morphological profiling to evaluate long-term visual prognosis following inverted ILM-flap macular hole surgery.

## 2. Materials and Methods

### 2.1. Study Design

This retrospective, consecutive case series included 40 patients (42 eyes) who underwent full-thickness macular hole surgery using the inverted flap technique at the 1st Department of Ophthalmology, Pomeranian Medical University in Szczecin, Poland. This study was conducted in accordance with institutional guidelines and the principles of the Declaration of Helsinki. The Bioethical Commission of Pomeranian Medical University reviewed the study protocol and determined that formal ethical approval was not needed because the research involved retrospective analysis of anonymised patient data. The exclusion criteria comprised conditions that could affect the objectivity of the study outcomes, such as other macular pathologies (e.g., age-related macular degeneration and diabetic macular oedema), glaucoma, prior vitrectomy, and a history of ocular trauma. Axial length (AXL) values were available from routine pre-cataract biometry records and were included in the dataset. No eyes with high myopia or myopia-related macular pathology were present among the included cases. All surgeries were performed between April 2022 and July 2024 by a single experienced vitreoretinal surgeon (A.M.). Every patient was pseudophakic at the time of the surgery. All ophthalmological interventions were performed using standard 25-gauge pars plana vitrectomy. During the operation, PVD was induced with the assistance of triamcinolone acetate. The ILM was peeled using brilliant blue–green (BBG) staining. ILM peeling was performed using the inverted ILM flap technique. At the end of the surgery, air-fluid exchange was performed, followed by SF6 tamponade. After the treatment, the patients were asked to hold a face-down position for a few days. Two patients were excluded from the study because of failure of macular hole closure following the initial surgery. However, both subsequently underwent a second pars plana vitrectomy, which resulted in successful closure. All the subjects underwent ophthalmic examinations before PPV and 12 months after the intervention. Best-corrected visual acuity (BCVA) was assessed at each visit using both ETDRS and Snellen charts. All measurements were performed by the same trained examiner under standardised lighting conditions and using the same patient-screen distance. The initial visit also included OCT analysis, microperimetry, and multifocal electroretinography (mfERG).

### 2.2. OCT Analysis

Spectral-domain optical coherence tomography (SD-OCT) was performed using the Heidelberg Spectralis OCT system (Heidelberg Engineering, Heidelberg, Germany). All scans were acquired using the IR and OCT mode with a 30° horizontal field of view (corresponding to approximately 8.8 mm of retinal coverage). The scans were obtained in high-speed mode (HS) with enhanced depth imaging (EDI). An automatic real-time (ART) function was applied to improve image quality. Only scans with a signal quality score of 25 or higher were included in the analysis. Infrared reflectance images were acquired simultaneously with OCT scans to assist in precise retinal positioning.

All the measurements were performed using the built-in calibre function of the OCT device. The data were acquired from cross-sectional images, which were taken closest to the macular hole centre. At each visit, the following parameters were measured: minimum linear diameter (MLD), which is the narrowest horizontal width of the macular hole; base diameter (BD), which is the width at the level of the retinal pigment epithelium; hole height (HH), representing the vertical distance from the RPE to the apex of the hole; hole left and right arm length; retinal thickness, representing central and parafoveal retinal thickness; and presence of focal RPE elevations.

**Figure 1:**
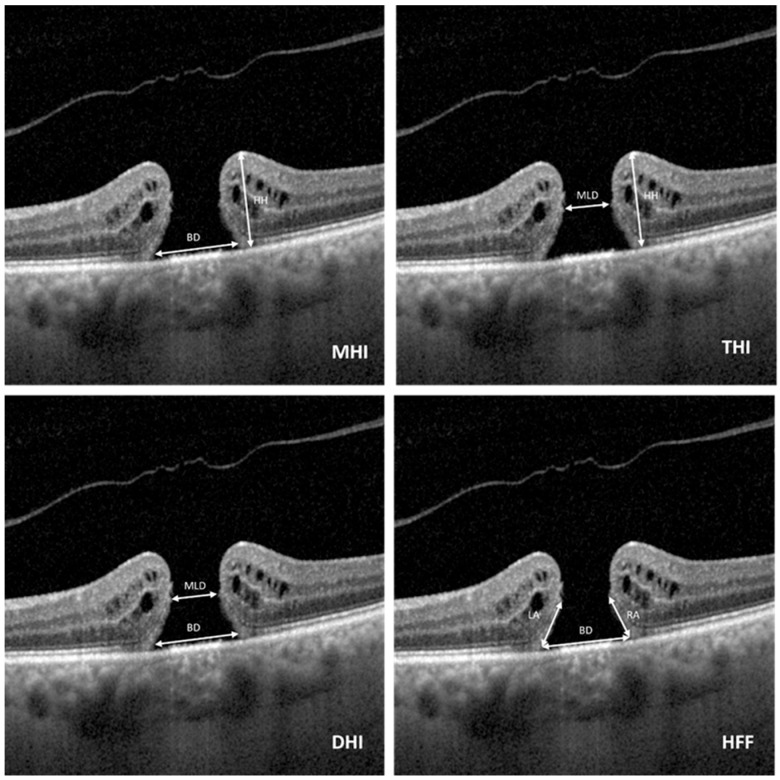
Schematic representation of the measured preoperative OCT parameters. All measurements were performed at the cross-section showing the largest macular hole (MH) diameter. The scans illustrate the following indices: macular hole index (MHI), tractional hole index (THI), diameter hole index (DHI), and hole form factor (HFF).

From the previously mentioned parameters, we calculated specific indices, such as the macula hole index (MHI), which is the maximum height/base diameter; tractional hole index (THI), which is maximum height/minimum hole diameter; diameter hole index (DHI), representing the minimum/maximum hole diameter; and hole form factor (HFF), which is the quotient of the sum of the left and right arm lengths divided by the base hole diameter (Figure 1). Vitreous adherence status was assessed and classified as follows: stage 1: complete adherence with a premacular bursa; stage 2: vitreomacular traction (VMT) with foveal adherence; stage 3: partial detachment over the macula with persistent attachment at the optic disc and peripheral retina; and stage 4: complete detachment involving the entire posterior pole.

Choroidal delineation was performed using the built-in calliper tool on spectral-domain OCT scans to measure subfoveal choroidal thickness and evaluate its longitudinal changes following full-thickness macular hole (FTMH) surgery. Measurements were obtained at baseline and during postoperative follow-up at standardised time points.

### 2.3. Multifocal Electroretinography (mfERG)

Multifocal electroretinography (RetiScan System, Roland Consult, Brandenburg an der Havel, Germany) examinations were carried out using the standard protocol routinely applied in our department, in accordance with ISCEV guidelines. The methodological details of this protocol have been previously reported by Ziontkowska-Wrzałek et al. [8]. Briefly, examinations were performed under full pharmacological dilation, with signals recorded using DTL fibre electrodes and a hexagonal stimulus array. Response amplitudes and implicit times were automatically derived for each concentric ring, and only recordings meeting ISCEV quality criteria were included in the analysis.

### 2.4. Microperymetry

Fundus-tracked microperimetry was conducted using a MAIA microperimeter (Centervue, Padova, Italy) following the routine protocol implemented in our department and previously detailed by Ziontkowska-Wrzałek et al. [8]. A 4–2 threshold strategy was applied using a 37-stimulus grid centred on the fovea. Retinal sensitivity metrics and fixation stability indices (P1, P2) were automatically generated by the system’s software (MAIA software version 2.6.0, Centervue, Padova, Italy).

### 2.5. Statistical Analysis

Qualitative variables were compared between groups with chi-square or Fisher’s exact tests. Because the distributions of most quantitative variables were significantly different from a normal distribution, the nonparametric Mann–Whitney test was used for analysis. A logistic regression model was used for multivariate analysis of potential predictors of postoperative BCVA > 0.5. Odds ratios (ORs) and 95% confidence intervals (95% CIs) were calculated for comparisons of the BCVA > 0.5 group to the BCVA ≤ 0.5 group. OR values for quantitative parameters reflected odds ratios of postoperative BCVA > 0.5 associated with an increase in a parameter by one unit (continuous variables) or one class (rank variables). *p* < 0.05 was considered to indicate statistical significance.

## 3. Results

### 3.1. Preoperative Morphological and Functional Parameters Associated with a Final BCVA > 0.5

Among the study subjects, 22 patients presented with a BCVA >0.5, and 20 had a BCVA ≤ 0.5 at the final visit. Demographic information, patient-reported symptoms, biometric data and baseline visual acuity data are summarised in Table 1. Patient age and sex were not associated with final visual outcome. Similarly, the presence of metamorphopsia was not associated with final visual acuity (95.5% in the BCVA > 0.5 group and 90% in the BCVA ≤ 0.5 group; *p* = 0.6). Axial length (AXL), which was retrospectively retrieved from pre-cataract biometry records, also did not differ significantly between the two groups (*p* = 0.341). Notably, patients with shorter disease durations were more likely to have a BCVA > 0.5 at follow-up (*p* = 0.002). Accordingly, patients with better initial visual scores were also more likely to develop BCVA > 0.5 postoperatively (*p* = 0.01).

Next, we attempted to identify specific SD-OCT-derived macular hole characteristics that were significantly associated with final visual outcomes (Table 2). We found a significant difference in well-defined structural parameters between the postoperative BCVA > 0.5 and BCVA ≤ 0.5 groups, including the MH area (*p* < 0.001), minimum MH diameter (*p* < 0.001), maximum MH diameter (*p* = 0.003), MHI (*p* < 0.001), THI (*p* < 0.001), DHI (*p* = 0.03) and HFF (*p* < 0.001). In addition to group comparisons, Spearman’s correlation analysis revealed significant associations between several OCT-derived parameters and final visual acuity. Parameters such as the MH area (Rs = −0.463; *p* = 0.0058), minimum MH diameter (Rs = −0.499; *p* = 0.0027), and maximum MH diameter (Rs = −0.397; *p* = 0.020) were negatively correlated with the final BCVA. These results indicate that larger hole dimensions were associated with poorer visual outcomes. Conversely, MHI (Rs = 0.525; *p* = 0.0014), THI (Rs = 0.538; *p* = 0.0010), and HFF (Rs = 0.499; *p* = 0.0027) were positively correlated with the final BCVA, suggesting that favourable macular hole configurations are related to better postoperative vision. These results support the importance of direct morphometric parameters and derived indices from preoperative OCT measurements in the estimation of visual recovery following FTMH surgery.

Interestingly, no relationship was noted between postoperative outcome and MH height (*p* = 0.801), vitreous adherence status (*p* = 0.078), initial retinal volume or perifoveal thickness. No statistically significant differences in preoperative choroidal thickness were observed between patients with a final BCVA ≤ 0.5 and those with a BCVA > 0.5.

In terms of initial microperimetry parameters, the group with a postoperative BCVA > 0.5 had significantly higher retinal sensitivity values compared with the group with a BCVA ≤ 0.5 (*p* = 0.03). However, initial microperimetry characteristics, such as detailed parameters of fixation stability and variability, did not significantly affect the final BCVA (Table 3). Similarly, neither the initial P-wave amplitudes in mfERG nor their culmination times were significantly associated with the final visual outcome when the BCVA > 0.5 and BCVA ≤ 0.5 groups were compared (Table 4). Interestingly, Spearman correlation analysis revealed a positive correlation between the final BCVA and preoperative mfERG amplitude: R1 ring amplitude (Rs = +0.36, *p* = 0.038) and R2 ring amplitude (Rs = +0.38, *p* = 0.028).

**Figure 2:**
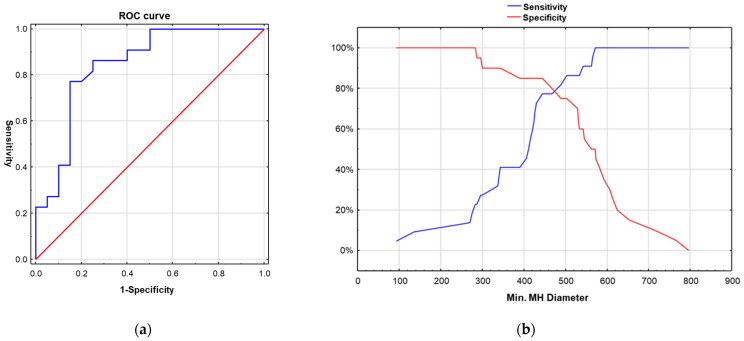
(**a**) ROC curve illustrating the predictive value of the minimum macular hole diameter for postoperative BCVA > 0.5. (**b**) Sensitivity and specificity of minimum macular hole diameter in predicting postoperative BCVA > 0.5.

Multivariate logistic regression analysis revealed both a smaller minimum macular hole (MH) diameter (OR = 0.98; 95% CI: 0.97–0.99; *p* = 0.004) and a shorter disease duration (OR = 0.11 per category step; 95% CI: 0.02–0.53; *p* = 0.005) as independent predictors of long term postoperative BCVA > 0.5. ROC analysis was performed exclusively for the continuous minimum MH diameter variable, confirming its strong discriminatory ability in predicting BCVA > 0.5 (AUC = 0.844; 95% CI: 0.721–0.967; *p* < 0.001) (Figure 2a) and identifying an optimal cut-off at approximately 470 µm (Figure 2b). Disease duration, although a significant prognostic factor in the regression model, was not included in the ROC analysis because of its categorical nature.

### 3.2. Relationships Between Baseline Macular Parameters and BCVA Change After Surgery

In the next step, we evaluated the potential relationships between preoperative macular parameters and the extent of BCVA change after surgery. We found that preoperative HFF was positively correlated with postoperative BCVA change (Rs = +0.39; *p* = 0.024). These results indicate that the greater the HFF before surgery is, the greater the positive change in BCVA postoperatively is. Accordingly, we found positive correlations between baseline central macular thickness in the central ETDRS field and changes in BCVA (Rs = +0.44; *p* = 0.01). These findings indicate that the greater the central macular area is, the greater the postoperative positive BCVA change. No significant correlations were found between BCVA change and the remaining preoperative OCT parameters. When analysing functional parameters, we found that the baseline P1 wave amplitude in the central ring of the multifocal electroretinogram (mfERG) was positively correlated with postoperative BCVA gain (Rs = +0.53; *p* = 0.0011). Significant correlations were also observed between BCVA gain and both the preoperative P1/P4 amplitude ratio (Rs = +0.479; *p* = 0.0036) and the P1/P5 ratio (Rs = +0.434; *p* = 0.0092). These results imply that patients with better preoperative retinal function (as indicated by higher central P wave amplitude) tend to experience greater visual improvement one year after FTMH surgery. Importantly, the BCVA change correlated negatively with the duration of MH (Rs = −0.41; *p* = 0.017). This indicates that a smaller improvement in BCVA would be expected in patients with a longer MH duration.

### 3.3. Associations Between Morphological and Functional Preoperative Hole Parameters

We observed that macular hole morphometric features on SD-OCT strongly corresponded with baseline macular function. Our analysis revealed negative associations between preoperative BCVA and keyhole dimensions, e.g., MH area (Rs = −0.512; *p* = 0.00053), minimum MH diameter (Rs = −0.411; *p* = 0.0069), and maximum MH diameter (Rs = −0.441; *p* = 0.0034), indicating that larger holes were associated with worse visual acuity at baseline. Conversely, positive correlations were observed between initial BCVA and MHI (Rs = 0.488, *p* = 0.0011) and THI (Rs = 0.355, *p* = 0.021), suggesting that more favourable macular hole configurations were associated with better baseline acuity. The maximum diameter of the full-thickness macular hole correlated negatively with preoperative fixation stability within a 4° diameter circle (Rs = −0.31; *p* = 0.047) and positively with 63% vertical fixation dispersion (Rs = +0.31; *p* = 0.047). Similarly, initial retinal sensitivity was negatively correlated with the MH area (Rs = −0.50; *p* < 0.001), minimum MH diameter (Rs = −0.54; *p* = <0.001), maximum MH diameter (Rs = −0.78; *p* = <0.001), and MH height (Rs = −0.56; *p* < 0.001) but positively correlated with the MHI (Rs = 0.60; *p* < 0.001) and THI (Rs = 0.35; *p* = 0.021). These findings underscore a consistent structure–function relationship, whereby unfavourable macular morphology is associated with reduced retinal function even before surgical intervention.

## 4. Discussion

In this study, we comprehensively evaluated the ability of preoperative anatomical and functional parameters, including OCT-derived morphometric measurements, microperimetry findings, and multifocal electroretinography (mfERG) results, to predict long-term visual outcomes following FTMH repair using the inverted-flap technique.

The factors most closely associated with satisfactory visual outcomes in our study were the minimum diameter of the macular hole and the duration of the macular hole before surgery. These findings align with those of previous studies demonstrating better visual recovery in eyes with smaller macular holes [9,10,11,12,13]. Importantly in our study axial length did not differ significantly between the two groups indicating that ocular magnification effects related to AXL, previously highlighted as a source of measurement error by Scoles and Mahmoud [14] were unlikely to influence our visual outcomes. In our model’s sensitivity–specificity analysis, 470 µm was identified as the optimal cut-off value, offering the best trade-off between sensitivity and specificity for predicting functional visual improvement after surgery. This aligns with the findings of Gupta et al., who identified a cut-off point of approximately 500 μm for achieving visual success, defined as reaching a BCVA of >0.5 [9]. Accordingly, in a large cohort study by Steel et al., a minimum linear diameter of approximately 500 μm marked the threshold at which the success rate began to decline [4]. In light of the above, we believe that the current MH classification proposed by Gass [15] and followed by the International Vitreomacular Traction Study Group (IVTS) [16], with a cut-off of 400 µm for large macular holes, is limited because it concentrates on rates of anatomical closure and does not reflect the true probability of functional success. Indeed, Satler et al. reported an excellent surgical prognosis for MHs with mid-hole diameters less than 500 microns. Ch’ng and colleagues reported a reduction in the success rate to approximately 75% for MHs with an MLD of 630 µm [17]. Accordingly, CLOSE Study Group Members [18] and other recent evidence propose simplifying the classification by defining a cut-off for good prognosis at 500 µm [4,9,17,19], as this more accurately represents the threshold above which the likelihood of successful closure with favourable visual outcomes begins to decline.

Additionally, our results reinforce previous observations that the geometry of macular holes plays a crucial role in postoperative visual recovery. We found that the values of key derived indices—MHI, THI, DHI, and HFF—were significantly different between favourable and unfavourable outcome groups. Moreover, MHI, THI, and HFF were positively correlated with final visual acuity, highlighting their prognostic value in predicting postoperative visual outcomes following FTMH surgery. The first study to use OCT for preoperative MH analysis was published by Ip et al. in 2002 [20]. Since then, numerous studies have described the importance of MH measurements and derived indices such as the HFF, MHI, DHI, and THI in the preoperative prediction of both anatomical closure and visual gain after MH repair surgery [11,21,22,23]. Our findings are consistent with those of Kusuhara et al. [21], who proposed MHI ≥ 0.5 as a benchmark for achieving favourable visual outcomes more than 20 years ago.

Additionally, our analysis revealed that the BCVA > 0.5 group presented with a significantly shorter disease duration, strongly indicating that longer-standing macular holes were associated with a diminished potential for visual recovery. This observation was corroborated in a large prospective cohort study by Steel et al. that reported that a shorter duration of symptoms was a crucial prognostic factor for visual success at the multivariate level [4]. Indeed, a meta-analysis conducted by Murphy et al. reported that each additional month of symptom duration is associated with decreased odds of primary hole closure and a decrease in postoperative BCVA [24]. The deleterious effect of prolonged MH duration may be attributed to progressive photoreceptor degeneration and retinal remodelling, compromising the capacity of the retina to achieve functional recovery even after successful anatomical closure [6]. In contrast, Stene-Johansen et al. reported that visual outcome was not dependent upon the duration of disease, with no significant difference in outcomes based on the duration of symptoms [25].

Considering the above findings, we searched for more objective functional tests to better predict visual outcomes in our patients. Given that mfERG responses are predominantly generated by cone photoreceptors, we evaluated their ability to predict functional success after macular hole repair. Indeed, mfERG served as a tool for measuring retinal function and predicting visual outcomes, e.g., post-PRP in eyes with early PDR [26]. Similarly, in terms of retinal toxicity (e.g., hydroxychloroquine retinopathy), central serous chorioretinopathy, epiretinal membrane, and age-related macular degeneration, multifocal electroretinography (mfERG) has demonstrated clinical utility in predicting treatment outcomes [27,28,29]. The central ring of the mfERG field map is correlated with structural OCT abnormalities at the corresponding points of the thickness map and BCVA [30]. Therefore, mfERG and OCT findings can complement one another to estimate visual acuity. A single report suggested that retinal function assessed using the ratio between central and peripheral mfERG responses could potentially be used as a predictor of visual acuity outcomes after macular hole surgery [31]. Indeed, Yolanda et al. reported that the diameter of the macular hole correlated with the severity of retinal dysfunction as assessed by both mfERG and visual acuity [32]. We found that both the preoperative R1 amplitude and the P1 amplitude ratio were significantly associated with postoperative BCVA gain. These findings suggest that eyes with relatively preserved central mfERG responses before surgery—likely reflecting the presence of functioning retinal cells within or around the macular hole—are more likely to experience meaningful visual improvement after surgery. However, we found that mfERG-derived functional parameters, including initial P wave amplitudes and peak times, did not significantly differ between BCVA outcome groups. Similar results have also been reported in previous research [33]. This may be explained by differences in how the size of the central mfERG stimulus and the extent of the macular hole affect retinal signal generation and visual acuity. In addition, it suggests that although mfERG may reflect macular function, it lacks the ability to predict surgical success.

Accordingly, we found that initial microperimetry parameters, particularly retinal sensitivity, differed significantly between eyes with BCVA >0.5 and those with BCVA ≤ 0.5. These findings likely reflect the close relationship between structure and function in full-thickness macular holes. Similarly, Borgia et al. and Bonnabel et al. reported that preoperative macular sensitivity parameters may serve as reliable predictors of postoperative BCVA [34,35]. Indeed, reduced retinal sensitivity indicates greater disruption of outer retinal layers and is strongly associated with impaired foveal function [36,37,38].

Furthermore, our study revealed significant correlations between preoperative retinal sensitivity and the area, minimum and maximum widths, and height of the macular hole, revealing that structural alterations of the macula directly limit functional retinal performance, even in the preoperative stage. Our results revealed that a larger maximum macular hole diameter was associated with poorer fixation stability and greater vertical fixation dispersion. These findings are consistent with those of previous studies reporting that fixation control deteriorates with increasing hole size, as larger defects disrupt more central photoreceptors and force the use of eccentric, less stable preferred retinal loci [39,40,41]. Indeed, the maximum hole diameter emerges as a key morphological determinant of preoperative fixation behaviour.

### 4.1. Limitations

This study has several limitations. The sample size was relatively small, which reduces statistical power and may limit the generalizability of the findings. The retrospective design also introduces potential selection bias and restricted access to certain postoperative structural parameters commonly used in current macular hole classification systems. Furthermore, many quantitative variables did not follow a normal distribution, necessitating the use of non-parametric tests, which are more conservative and may reduce sensitivity to detect weaker associations. Another limitation is the absence of direct postoperative correlation with established structural biomarkers, such as restoration of the external limiting membrane or ellipsoid zone. Because the primary aim of the study was to identify preoperative anatomical and functional predictors of visual outcome, postoperative parameters were not included in the analysis. Finally, multimodal functional testing (microperimetry and mfERG) requires standardized acquisition conditions and specialized equipment, which may limit the applicability of these methods in clinical settings.

Despite these constraints, the consistent associations observed between structural and functional measurements support the credibility of the overall findings.

### 4.2. Future Directions

The multimodal data collected in this study also point toward promising opportunities for automated analysis. Future research could explore machine learning or deep learning models capable of combining multiple preoperative structural and functional parameters to enhance the prediction of postoperative visual acuity. In addition, OCT-based algorithms may help standardize the assessment of macular hole configuration and closure. As these technologies advance, they may provide clinicians with more objective and efficient tools to support surgical planning and prognostic evaluation.

## 5. Conclusions

This study demonstrates that preoperative macular hole size and symptom duration constitute the principal independent predictors of long-term functional visual recovery following FTMH repair using the inverted-flap technique. Our sensitivity–specificity analysis identified an optimal prognostic threshold of approximately 470 µm to predict postoperative BCVA > 0.5. Similarly, a shorter duration of symptoms independently increased the probability of reaching a favourable visual outcome, emphasizing the clinical importance of timely diagnosis and early surgical intervention.

In addition to these primary determinants, several OCT-derived morphometric indices—including MHI, THI, DHI, and HFF—demonstrated significant associations with postoperative visual acuity. These findings suggest that favourable macular hole geometry provides structural conditions that facilitate more effective retinal recovery. Functional assessments offered complementary prognostic value: higher preoperative retinal sensitivity on microperimetry and greater central mfERG amplitudes correlated with larger postoperative improvements in BCVA, indicating that preserved macular function prior to surgery enhances the potential for visual restoration, even when anatomical closure is achieved.

Overall, these findings underscore that both anatomical (minimum diameter, geometrical indices) and functional (retinal sensitivity, mfERG measures) preoperative parameters offer clinically meaningful prognostic information. This supports the rationale for incorporation of a multimodal, structure–function-based assessment into preoperative evaluation protocols for patients undergoing FTMH surgery.

## Figures and Tables

**Table 1:** Comparison of baseline demographic and clinical features between eyes with postoperative BCVA > 0.5 and ≤0.5. All BCVA values refer to baseline (preoperative) measurements.

Parameter	BCVA > 0.5 Group	BCVA ≤ 0.5 Group	*p* *
Age Median (IQR)	69 (5)	69 (6.5)	0.850
Gender (F/M)	15/7	18/2	0.135
Duration of disease, [n] (%)			
<6 M	15 (68.2%)	5 (25%)	0.002
6–12 M	6 (27.3%)	8 (40%)	
>12 M	1 (4.5%)	7 (35%)	
BCVA [Snellen charts] Median (IQR)	0.2 (0.04)	0.1 (0.155)	0.015
BCVA [ETDRS charts] Median (IQR)	3.5 (6)	1 (3)	0.004
Axial length [mm] Median (IQR)	22.97 (2.33)	22.91 (1.25)	0.341

* Fisher’s exact test for categorical variables or the Mann-Whitney U test for continuous and ordinal variables. IQR, interquartile range.

**Table 2:** Comparison of preoperative OCT-derived parameters between eyes with postoperative BCVA > 0.5 and ≤0.5 after FTMH surgery.

Parameter	BCVA > 0.5 Group Median (IQR)	BCVA ≤ 0.5 Group Median (IQR)	*p* *
MH area [mm^2^]	0.155 (0.12)	0.295 (0.085)	<0.001
Min. MH diameter [μm]	413 (148)	567.5 (110)	<0.001
Max. MH diameter [μm]	968 (375)	1154.7 (244.5)	0.003
MH height [μm]	470.5 (176)	474.2 (46.5)	0.801
MHI	0.51 (0.15)	0.41 (0.07)	<0.001
THI	1.31 (0.3)	0.88 (0.22)	<0.001
DHI	0.41 (0.13)	0.47 (0.08)	0.030
HFF	0.79 (0.15)	0.65 (0.12)	<0.001
RPE elevations (%)	59%	50%	0.757
Total retinal volume [mm^3^]	9.385 (0.84)	9.36 (0.62)	0.410
ETDRS retinal thickness Central [μm]	408.5 (96)	350 (59)	0.017
ETDRS retinal thickness Inner Superior [μm]	398.5 (85)	428 (78)	0.539
ETDRS retinal thickness Inner Nasal [μm]	421.5 (93)	427 (68)	0.990
ETDRS retinal thickness Inner Inferior [μm]	406 (75)	427 (68)	0.388
ETDRS retinal thickness Inner Temporal [μm]	406.5 (49)	418 (76)	0.488
ETDRS retinal thickness Outer Superior [μm]	307.5 (34)	306 (32)	0.505
ETDRS retinal thickness Outer Nasal [μm]	325 (34)	311 (35)	0.027
ETDRS retinal thickness Outer Inferior [μm]	299.5 (28)	294 (31)	0.061
ETDRS retinal thickness Outer Temporal [μm]	299.5 (29)	288 (33)	0.150
Choroidal thickness [μm]	236 (70)	229.5 (157)	0.529
Vitreomacular traction [n] (%)			
0	3 (13.6%)	0 (0%)	0.078
1	7 (31.8%)	4 (20%)	
2	8 (36.4%)	10 (50%)	
3	4 (18.2%)	6 (30%)	

* Fisher’s exact test for categorical variables or the Mann-Whitney U test for continuous and ordinal variables. IQR, interquartile range.

**Table 3:** Comparison of baseline microperimetric parameters between eyes with postoperative BCVA > 0.5 and ≤0.5.

Parameter	BCVA > 0.5 Group Median (IQR)	BCVA ≤ 0.5 Group Median (IQR)	*p* *
Average Threshold [dB]	23.25 (3.6)	21.35 (3.45)	0.03
Fixation Stability P1 [%]	89 (21)	88.5 (32)	0.980
Fixation Stability P2 [%]	99.5 (5.5)	100 (3)	0.765
63% BCEA: horizontal [°]	1.55 (0.7)	1.45 (1.2)	0.93
63% BCEA: vertical [°]	1.1 (1.7)	1.2 (0.95)	0.63
63% BCEA: area [deg^2^]	1.25 (1.4)	1.4 (2.85)	0.88
95% BCEA: horizontal [°]	2.65 (1.3)	2.55 (2.1)	0.88
95% BCEA: vertical [°]	1.95 (2.8)	2.05 (1.65)	0.687
95% BCEA: area [deg^2^]	3.75 (4.1)	4.15 (8.45)	0.84

* Mann–Whitney U test. IQR, interquartile range.

**Table 4:** Comparison of P wave amplitude and culmination time across retinal rings (R1–R6) between eyes with postoperative BCVA > 0.5 and ≤0.5.

Parameter	BCVA > 0.5 Group Median (IQR)	BCVA ≤ 0.5 Group Median (IQR)	*p* *
P wave amplitude in R1 [nV/degree^2^]	51.575 (40.25)	35.61 (32.35)	0.087
P wave amplitude in R2 [nV/degree^2^]	46.58 (19.93)	40.15 (23.275)	0.07
P wave amplitude in R3 [nV/degree^2^]	31.445 (15.01)	29.37 (21.065)	0.80
P wave amplitude in R4 [nV/degree^2^]	22.525 (14.11)	26.605 (15.525)	0.65
P wave amplitude in R5 [nV/degree^2^]	17.715 (10.17)	18.65 (12.572)	0.78
P wave amplitude in R6 [nV/degree^2^]	16.05 (8.04)	14.74 (10.9)	0.61
P1 wave implicit time in R1 [ms]	45.6 (4.9)	44.105 (7.8)	0.69
P1 wave implicit time in R2 [ms]	42.2 (1.9)	42.65 (1.9)	0.35
P1 wave implicit time in R3 [ms]	41.2 (2)	41.2 (1.45)	0.3
P1 wave implicit time in R4 [ms]	41.2 (2)	41.2 (2.45)	0.51
P1 wave implicit time in R5 [ms]	41.7 (2.9)	41.7 (2.4)	0.80
P1 wave implicit time in R6 [ms]	41.2 (3.9)	41.2 (2)	0.97

* Mann–Whitney U test. IQR, interquartile range.

## Data Availability

The data that were used to support the findings of this study are. available from the corresponding author upon request.
